# The association of triglyceride-glucose and triglyceride-glucose related indices with the risk of heart disease in a national cohort study

**DOI:** 10.1186/s12933-025-02621-y

**Published:** 2025-02-06

**Authors:** Xiaodi Tang, Kexin Zhang, Rong He

**Affiliations:** Department of Cardiovascular Medicine, Beijing Tsinghua Changgung Hospital, School of Clinical Medicine, Tsinghua University, No. 168 Litang Road, Changping District, Beijing, China

**Keywords:** Triglyceride‑glucose index, Triglyceride-glucose related indices, Heart disease, Positive association, Diabetes

## Abstract

**Background:**

The association between triglyceride-glucose (TyG), triglyceride-glucose related indices and heart disease remains a topic of debate in the current literature. Existing evidence in Chinese people and in diabetes or non-diabetes patients regarding this association is limited, highlighting the need for further investigation.

**Methods:**

A cohort study was conducted involving 7945 participants enrolled in the China Health and Retirement Longitudinal Study (CHARLS). The incidence of heart disease was collected by following up 9 years. TyG, TyG with body mass index (BMI), waist circumference (WC), waist-to-height ratio (WHtR) were collected at baseline. Multivariate Cox proportional hazards models, restricted cubic spline (RCS), Kaplan-Meier (KM) curves, subgroup analysis and sensitivity analysis were used to analyze the associations between TyG, TyG related indices and the risk of heart disease in national participants and in type 2 diabetes (T2D) or non-T2D patients respectively.

**Results:**

During a 9-year follow-up, 1477 participants (18.6%) developed heart disease. Our analysis found a significant positive association between TyG-BMI, TyG-WC, and TyG-WHtR with heart disease risk in all participants. The adjusted hazard ratios (HR) for the highest quartile compared to the lowest were: TyG-BMI 1.73 (95% CI: 1.47–2.03), TyG-WC 1.46 (95% CI: 1.24–1.71), and TyG-WHtR 1.31 (95% CI: 1.11–1.54). However, TyG alone was not significantly associated with heart disease in all participants. In non-diabetic patients, the associations were consistent: TyG-BMI 1.75 (95% CI: 1.47–2.08), TyG-WC 1.47 (95% CI: 1.24–1.75), and TyG-WHtR 1.34 (95% CI: 1.13–1.60). However, in diabetes patients, no significant associations were found between TyG, TyG-WC, TyG-WHtR and heart disease risk except for the highest quartile of TyG-BMI (HR: 1.86, 95% CI: 1.02–3.40).

**Conclusions:**

In the national population study, higher TyG-BMI, TyG-WC, and TyG-WHtR indices were significantly associated with an increased risk of heart disease, with TyG-BMI and TyG-WC showing stronger correlations. While this association was evident in non-T2D patients, only TyG-BMI was associated with heart disease incidence in T2D patients, underscoring the necessity for further investigation.

**Supplementary Information:**

The online version contains supplementary material available at 10.1186/s12933-025-02621-y.

## Introduction

The prevalence and mortality rates of cardiovascular diseases in China have continued to rise in recent years, becoming an important public health problem affecting the nation’s health [1]. A number of studies and reports indicate that approximately 330 million people in China are affected by cardiovascular diseases, representing approximately one-quarter of the national population. Among these diseases, stroke, heart disease, heart failure, and hypertension are particularly prevalent [2].

The link between insulin resistance (IR) and the development of cardiovascular disease (CVD) has now been corroborated by a number of studies. A number of studies have demonstrated that insulin resistance represents a significant independent risk factor for CVD [3]. The hyperinsulin-positive glucose clamp test is considered the gold standard for evaluating IR, but it is rarely used in the clinic due to its high cost, accessibility, and reproducibility. In this context, researchers have proposed various methods to evaluate IR [9], such as QUICKI, homeostasis model assessment of insulin resistance (HOMA-IR) and Bennett ISI index. Among these indicators, HOMA-IR is the most commonly used indicator in clinical Settings, but its calculation is complex and expensive. TyG index is considered to be a reliable alternative marker of IR. As an IR substitute index, TyG index is simple, economical and standardized, which is conducive to the implementation in clinical work and helps to early screening high-risk groups of vascular diseases [4]. Furthermore, its effects are not confined to diabetic patients, but are also observed in a broad range of non-diabetic populations [5, 6]. In recent years, the triglyceride-glucose related (TyG) indices, including TyG, TyG with body mass index (TyG-BMI), TyG with waist circumference (TyG-WC), and TyG with waist-to-height ratio (TyG-WHtR), have emerged as promising indicators of insulin resistance [7, 8]. Nevertheless, the accuracy of the TyG and TyG related indices in assessing insulin resistance varies depending on the ethnic and cultural context. For instance, TyG-BMI was proposed as a surrogate marker for the clinical assessment of insulin resistance in a study of Korean adults. A synthesis of the latest studies and related advances revealed that TyG-BMI and TyG-WC performed more prominently in assessing insulin resistance. TyG-WHtR, although slightly inferior in performance, still has some assessing value [9].

Nevertheless, the evidence supporting the association between TyG as well as TyG related indices and the incidence of cardiovascular disease in the Chinese population remains inconclusive [10]. Furthermore, the potential differences in the association between TyG as well as TyG related indices and cardiovascular events in type 2 diabetes (T2D) and non-diabetes populations have yet to be elucidated [11, 12]. Accordingly, the objective of this study is to examine the association between TyG and its related indices with cardiovascular risk in the national population, with additional focus on whether this relationship is consistent between T2D and non-T2D individuals [13].

## Methods

### Study design

The data used in this study were obtained from the China Health and Retirement Longitudinal Study (CHARLS), a nationally representative longitudinal survey that gathers information from individuals aged 45 years and above in China [14]. The survey encompasses a comprehensive range of data, including economic status, physical and psychological health, demographics, and social networks. Furthermore, the survey assesses anthropometric indicators and cardiometabolic diseases. The initial national baseline survey of CHARLS was conducted in 2011, with 17,705 individuals from 150 counties/districts and 450 villages/resident communities participating. Subsequently, participants were followed up every two years. This study was approved by the Biomedical Ethics Review Committee of Peking University, and all participants provided signed informed consent forms [15].

### Study population

**Fig. 1:**
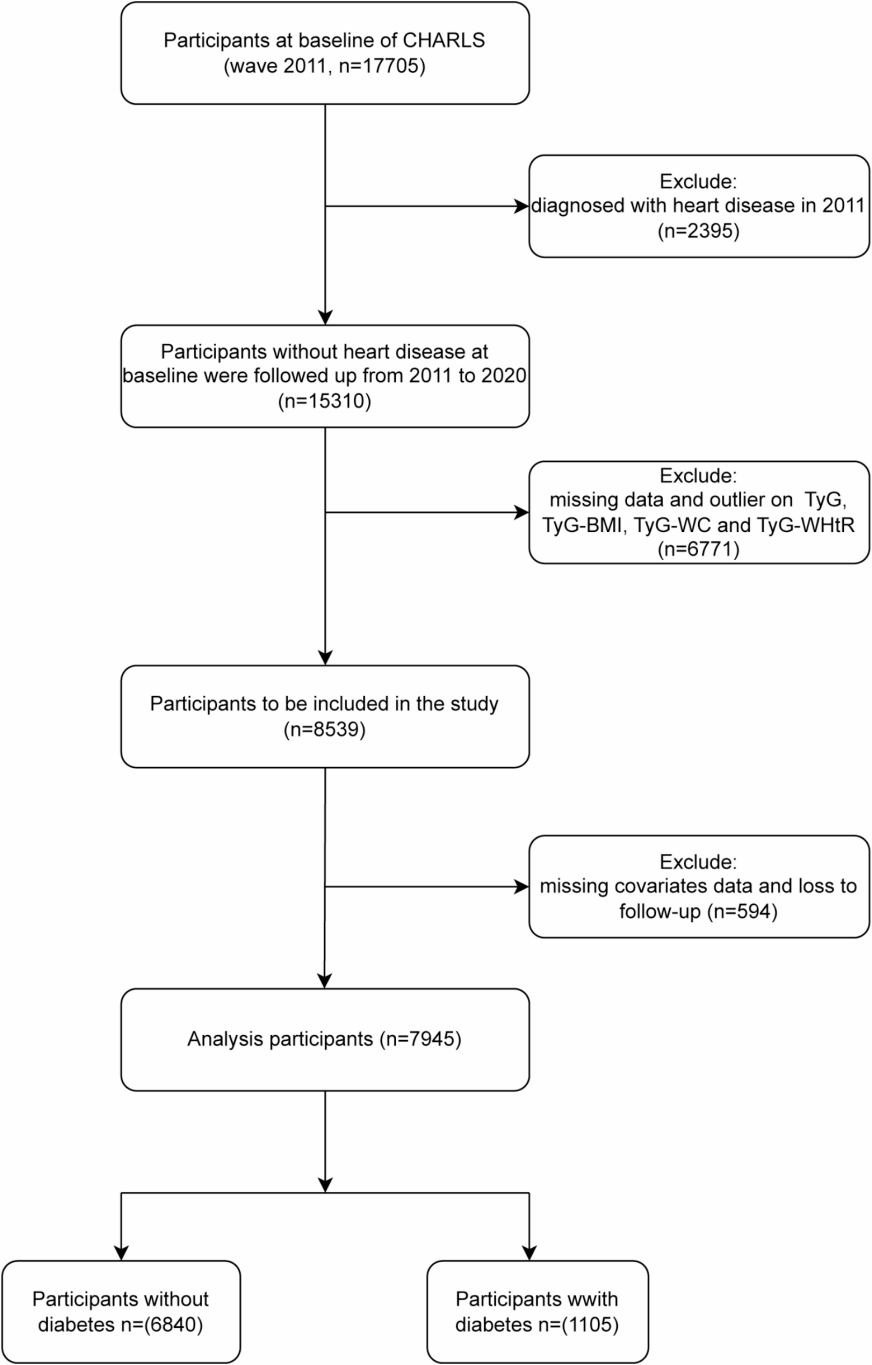
Flow chart of the study population inclusion

All participants recruited in the national baseline survey were included if they met the following criteria: (1) no history of heart disease at baseline; (2) complete collection of TyG and TyG related indices; (3) completion of follow-up. Ultimately, 7945 individuals without heart disease at baseline in 2011 were included (Fig. 1).

### TyG and TyG related indices

Expert medical staff obtained venous blood samples from fasting patients by following established protocols, and the analyses were conducted in a central laboratory. Serum triglyceride and fasting plasma glucose (FPG) were measured using an enzymatic colorimetric method. The TyG index was calculated using the formula: TyG index = ln(TG (mg/dL)× FPG (mg/dL) / 2), where TG was measured using a Roche Modular P and Cobas 6000 system, and FPG was determined by the hexokinase method using a Roche/Hitachi Cobas C 501 analyzer [16].

Height and weight are measured using a stadiometer and a weighing scale, with participants barefoot and in light clothing. When measuring waist circumference, trained measurers use a flexible tape measure to encircle the waist at the level of the navel. Height, weight, and waist circumference (WC) were physically measured three times, and the average values were adopted as the final measurements.

BMI is calculated as weight (kg) divided by the square of height (m). Waist-to-height ratio (WHtR) was defined as WC/height. TyG was modified by multiplying with BMI, WC, and WHtR to produce TyG-BMI, TyG-WC, and TyG-WHtR, respectively [17]. TyG index and TyG related indices were grouped into quartile (Q).

$$ \begin{gathered} {\text{TyG}} - {\text{BMI}} = {\text{TyG}} \times {\text{BMI}}. \hfill \\ {\text{TyG}} - {\text{WC}} = {\text{TyG}} \times {\text{WC}}. \hfill \\ {\text{TyG}} - {\text{WHtR}} = {\text{TyG}} \times {\text{WHtR}}. \hfill \\ \end{gathered} $$

### Outcome and follow-up

Heart disease was defined as self-reported diagnosis using standardized questions, as indicated by a positive response to the question “Have you been told by a doctor that you have been diagnosed with a heart attack, coronary heart disease, angina, heart failure, or other heart problems?” based on the CHARLS questionnaire [18]. Participants were followed from the baseline year 2011 until the onset of heart disease or the date of the last survey in 2020, whichever came first.

### Covariates

Covariates included social demographic characteristics, vital signs, lifestyle factors, laboratory tests and disease history. The information about demographic characteristics, lifestyle factors, disease history was collected using standardized questionnaires. Social demographic characteristics included age, sex, marital status and residence (rural/urban). Lifestyle factors included smoking status (ever smoking/never smoking) and alcohol status (ever drinking/never drinking). Disease history included hypertension, diabetes, cancer, lung disease, stroke, liver disease, kidney disease, digestive disease, asthma. Hypertension was defined as systolic blood pressure ≥ 140 mmHg and/or diastolic pressure ≥ 90 mmHg and/or self-reported diagnosis history of hypertension and/or antihypertensive medications currently used. Diabetes was defined as fasting glucose ≥ 7.0 mmol/L or self-reported diagnosis history of diabetes or use of any hypoglycemic medication currently used. Kidney disease was defined as estimated glomerular filtration rate (eGFR) < 60 ml/min/1.73 m² and/or self-reported diagnosis history. The measurement of vital signs consists mainly of pulse rate measurements, which are performed using an OMRON HEM7200 electronic sphygmomanometer. During the measurement, the subject needs to keep quiet and measure the pulse rate three times, each measurement interval of 45 s, take the average of the three measurement results as the final pulse rate [18].

Laboratory tests at baseline in 2011 included white blood cell count (WBC), mean corpuscular volume (MCV), platelet count, blood urea nitrogen (BUN), glucose, serum creatinine (Scr), total cholesterol (TC), triglycerides (TG), high-density lipoprotein (HDL) cholesterol, low-density lipoprotein (LDL) cholesterol, C-reactive protein (CRP), glycosylated haemoglobin (HbA1c), uric acid (UA), haematocrit (HCT), haemoglobin (HGB). Blood samples are collected by medical professionals at the site of the investigation and immediately sent to the laboratory of the local partner hospital for analysis. The laboratory adopts standardized testing methods and equipment to ensure the accuracy and reliability of data.

### Statistical analysis

All normally distributed continuous variables were reported as mean ± standard deviation (SD), while categorical variables were presented as percentages. Group comparisons for continuous variables were conducted using either the independent samples Student’s t-test or the Mann-Whitney U-test, depending on the distribution’s normality. Categorical data were compared using the chi-square test as appropriate.

Multivariate Cox regression models were employed to calculate hazard ratios (HRs) and their corresponding 95% confidence intervals (CIs) for the heart disease risk. The assumption of proportional hazards was evaluated by examining “log-log” plots and by testing interactions with survival time. TyG and TyG related indices was analyzed as continuous and categorical variables divided into quartile respectively. The selection of potential confounding variables was guided by clinical relevance and existing scientific literature. Variables were also included if they led to a greater than 10% change in the effect estimate.

To explore potential linear dose-response associations between TyG, TyG related indices and heart disease, we utilized a restricted cubic spline (RCS) model to generate smooth curves. we further used a two-piecewise model to fit the link if there were non-linear relationship.

Kaplan-Meier (KM) curves were used to show the different survival patterns among the all participants with different quartiles of the TyG index and TyG related indices.

The handling of missing data involved list-wise deletion, as missing values accounted for less than 5% of the dataset. Subgroup analyses were performed to assess the robustness of the study findings. E-value values were tested for the influence of unmeasured confounders as a sensitivity analysis.

All statistical analyses were conducted using R Statistical Software (Version 4.2.2, http://www.R-project.org, The R Foundation) and Free Statistics Analysis Platform (Version 1.9, Beijing, China, http://www.clinicalscientists.cn/freestatistics).

## Results

### Baseline characteristic of participants

Based on the inclusion and exclusion criteria (Fig. 1), We enrolled a total of 7945 patients with a mean age of 58.7 ± 9.6 years, of whom 3698 (46.5%) were male. Over a maximum follow-up period of 9.0 years, a total of 1477 (18.6%) participants experienced heart disease events. Table 1 presents the demographic characteristics of the participants categorized by TyG index. Participants with higher TyG index were found to be older, with a higher level of WBC, CRP and a higher prevalence of hypertension and diabetes (*P* < 0.001).

**Table 1:** Baseline characteristics of included subjects stratified by baseline TyG index

Variables	Total (*n* = 7945)	Q1 (*n* = 2001)	Q2 (*n* = 1976)	Q3 (*n* = 1980)	Q4 (*n* = 1988)	*p*
		5.18–8.21	8.21–8.58	8.58–9.02	9.01–13.03	
Age, years	58.7 ± 9.6	58.6 ± 10.0	58.8 ± 9.8	59.3 ± 9.6	58.3 ± 9.1	0.007
Male, n (%)	3698 (46.5)	1074 (53.7)	941 (47.6)	826 (41.7)	857 (43.1)	< 0.001
Marry, n (%)	7036 (88.6)	1767 (88.3)	1757 (88.9)	1741 (87.9)	1771 (89.1)	0.638
Rural, n (%)	5316 (66.9)	1444 (72.2)	1356 (68.6)	1301 (65.7)	1215 (61.1)	< 0.001
Smoke, n (%)	3080 (38.8)	858 (42.9)	792 (40.1)	727 (36.7)	703 (35.4)	< 0.001
Drink, n (%)	3126 (39.4)	861 (43)	797 (40.4)	702 (35.5)	766 (38.6)	< 0.001
WBC, 10^9/L	6.3 ± 2.2	6.0 ± 1.8	6.2 ± 1.9	6.4 ± 3.0	6.6 ± 1.9	< 0.001
MCV, fl.	90.6 ± 8.6	90.8 ± 9.3	90.4 ± 8.8	90.8 ± 8.6	90.6 ± 7.8	0.352
Platelet, 10^9/L	212.4 ± 75.9	210.5 ± 73.8	210.5 ± 73.7	213.1 ± 83.1	215.2 ± 72.7	0.151
BUN, mg/dl	15.7 ± 4.6	16.4 ± 5.0	15.6 ± 4.5	15.4 ± 4.3	15.4 ± 4.3	< 0.001
Glucose, mg/dl	109.9 ± 36.7	95.4 ± 13.4	101.4 ± 14.4	107.1 ± 19.4	135.5 ± 60.5	< 0.001
Scr, mg/dl	0.8 ± 0.2	0.8 ± 0.2	0.8 ± 0.3	0.8 ± 0.2	0.8 ± 0.2	0.071
TC, mg/dl	193.3 ± 38.4	178.8 ± 33.2	189.6 ± 33.8	196.8 ± 36.3	207.9 ± 43.5	< 0.001
TG, mg/dl	103.5 (74.3, 150.4)	61.1 (52.2, 69.0)	88.5 (79.7, 99.1)	125.7 (110.6, 141.6)	205.3 (169.0, 272.8)	< 0.001
HDL, mg/dl	51.4 ± 15.2	60.3 ± 15.2	54.8 ± 13.6	49.4 ± 13.0	41.1 ± 11.9	< 0.001
LDL, mg/dl	116.2 ± 34.7	108.2 ± 29.3	118.7 ± 30.9	123.4 ± 33.9	114.4 ± 41.7	< 0.001
CRP, Median	1.0 (0.5, 2.1)	0.8 (0.4, 1.8)	0.9 (0.5, 1.9)	1.0 (0.6, 2.2)	1.3 (0.7, 2.5)	< 0.001
HbA1c, %	5.3 ± 0.8	5.1 ± 0.4	5.1 ± 0.5	5.2 ± 0.6	5.6 ± 1.3	< 0.001
UA, mg/dl	4.4 ± 1.2	4.2 ± 1.1	4.3 ± 1.2	4.4 ± 1.3	4.7 ± 1.4	< 0.001
HCT, %	41.6 ± 6.1	41.1 ± 6.2	41.2 ± 6.1	41.6 ± 6.1	42.5 ± 5.9	< 0.001
HGB, g/dl	14.3 ± 2.2	14.1 ± 2.3	14.3 ± 2.2	14.4 ± 2.2	14.7 ± 2.2	< 0.001
CysC, mg/l	1.0 ± 0.3	1.0 ± 0.3	1.0 ± 0.3	1.0 ± 0.3	1.0 ± 0.3	< 0.001
Hypertension, n (%)	3635 (45.8)	712 (35.6)	834 (42.2)	981 (49.5)	1108 (55.7)	< 0.001
Diabetes, n (%)	411 ( 5.2)	30 (1.5)	58 (2.9)	87 (4.4)	236 (11.9)	< 0.001
Cancer, n (%)	67 ( 0.8)	12 (0.6)	18 (0.9)	13 (0.7)	24 (1.2)	0.138
Lung disease, n(%)	706 ( 8.9)	189 (9.4)	195 (9.9)	165 (8.3)	157 (7.9)	0.099
Stroke, n (%)	174 ( 2.2)	30 (1.5)	46 (2.3)	41 (2.1)	57 (2.9)	0.029
Liver disease, n (%)	238 ( 3.0)	74 (3.7)	50 (2.5)	57 (2.9)	57 (2.9)	0.166
Renal disease, n (%)	393 ( 4.9)	101 (5)	112 (5.7)	92 (4.6)	88 (4.4)	0.289
Digestive disease, n (%)	1691 (21.3)	460 (23)	472 (23.9)	371 (18.7)	388 (19.5)	< 0.001
Asthma, n (%)	318 ( 4.0)	88 (4.4)	84 (4.3)	77 (3.9)	69 (3.5)	0.447
BMI, kg/m²	23.4 ± 3.8	22.1 ± 3.2	22.9 ± 3.8	23.7 ± 3.7	24.9 ± 3.6	< 0.001

WBC: white blood cell count, MCV: mean corpuscular volume, BUN: blood urea nitrogen, Scr: Creatinine, TC: Total Cholesterol, TG: Triglycerides, HDL: high density lipoprotein cholesterol, LDL: low density lipoprotein cholesterol, CRP: C-Reactive Protein, HbA1c: glycated hemoglobin, UA: Uric Acid, HCT: Hematocrit, HGB: Hemoglobin, BMI: body mass index, WC: waist circumference

**Fig. 2:**
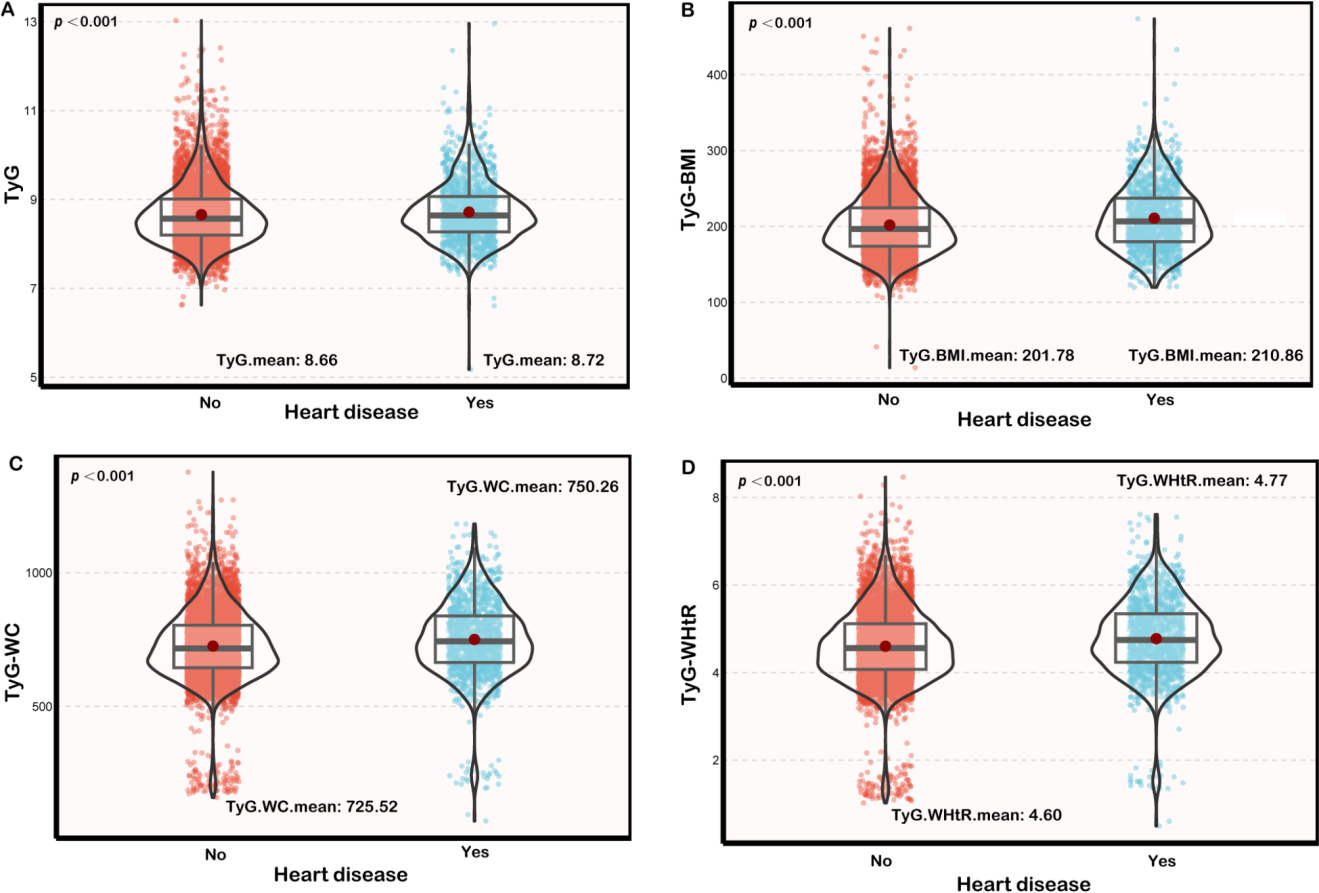
Difference in distribution of TyG (**A**), TyG-BMI (*B*), TyG-WC (**C**), TyG-WHtR (**D**) in heart disease and non-heart disease groups. The solid gray line in the middle of the Box Plot represents the median, and the upper and lower edge lines of the box plot represent the first quartile and the third quartile. The confluence of the two ends of the Violin Plot represents the lower adjusted value and upper adjacent value. The linear parts of the Violin Plot at the upper and lower ends reflect the outside points. The scatter plots in the red and blue sections visually reflect the distribution of data. TyG: Triglyceride and glucose, TyG-BMI: Triglyceride and glucose with body mass index, TyG-WC: Triglyceride and glucose with waist circumference, TyG-WHtR: Triglyceride and glucose with waist-to-height ratio

According to Fig. 2, the higher TyG and TyG related indicators were observed in heart disease patients as continuous variables (TyG. mean: 8.66 vs. 8.72; TyG-BMI. mean: 201.78 vs. 210.86; TyG-WC. mean: 725.52 vs. 750.26; TyG-WHtR. mean: 4.60 vs. 4.77).

### The association of TyG and TyG related indices with heart disease in all participants

The Kaplan–Meier curves of the cumulative incidence of heart disease were shown in Supplementary Material 1: Fig. S1. In the multivariable Cox proportional hazards model, after adjustment for potential confounders as shown in Table 2 (model 3), per SD increased of TyG-BMI, TyG-WC and TyG-WHtR were found to be significantly associated with an increased risk of heart disease (TyG-BMI, HR: 1.19, 95% CI: 1.13–1.25, *P* < 0.001; TyG-WC, HR: 1.15, 95% CI: 1.08–1.22, *P* < 0.001, TyG-WHtR, HR: 1.10, 95% CI: 1.03–1.16, *P* = 0.002). However, TyG was not found to be associated with the risk of heart disease (HR: 1.03, 95% CI: 0.97–1.09).

**Table 2:** The association of TyG, TyG-BMI, TyG-WC and TyG-WHtR with heart diseas in all participants

Variable	Model 1		Model 2		Model 3	
	HR (95%CI)	*P* value	HR (95%CI)	*P* value	HR (95%CI)	*P* value
TyG	1.07 (1.02–1.13)	0.008	1.07 (1.02–1.13)	0.009	1.03 (0.97–1.09)	0.362
Quartile						
Q1	1(Ref)		1(Ref)		1(Ref)	
Q2	1.11 (0.95–1.29)	0.175	1.09 (0.94–1.27)	0.244	1.05 (0.91–1.23)	0.495
Q3	1.14 (0.98–1.32)	0.082	1.15 (0.99–1.33)	0.07	1.07 (0.92–1.25)	0.355
Q4	1.21 (1.05–1.41)	0.01	1.21 (1.04–1.40)	0.013	1.08 (0.92–1.27)	0.33
P for trend		0.011		0.011		0.321
TyG-BMI	1.22 (1.16–1.28)	< 0.001	1.23 (1.17–1.29)	< 0.001	1.19 (1.13–1.25)	< 0.001
Quartile						
Q1	1(Ref)		1(Ref)		1(Ref)	
Q2	1.28 (1.09–1.5)	0.002	1.31 (1.12–1.53)	0.001	1.27 (1.08–1.49)	0.003
Q3	1.31 (1.12–1.54)	0.001	1.35 (1.16–1.59)	< 0.001	1.27 (1.08–1.49)	0.004
Q4	1.86 (1.60–2.16)	< 0.001	1.91 (1.64–2.23)	< 0.001	1.73 (1.47–2.03)	< 0.001
P for trend		< 0.001		< 0.001		< 0.001
TyG-WC	1.18 (1.12–1.25)	< 0.001	1.19 (1.13–1.26)	< 0.001	1.15 (1.08–1.22)	< 0.001
Quartile						
Q1	1(Ref)		1(Ref)		1(Ref)	
Q2	1.15 (0.98–1.34)	0.085	1.16 (0.99–1.35)	0.065	1.12 (0.96–1.32)	0.143
Q3	1.31 (1.13–1.53)	< 0.001	1.33 (1.14–1.56)	< 0.001	1.25 (1.07–1.46)	0.005
Q4	1.62 (1.39–1.87)	< 0.001	1.63 (1.40–1.90)	< 0.001	1.46 (1.24–1.71)	< 0.001
P for trend		< 0.001		< 0.001		< 0.001
TyG-WHtR	1.15 (1.09–1.21)	< 0.001	1.15 (1.09–1.22)	< 0.001	1.10 (1.03–1.16)	0.002
Quartile						
Q1	1(Ref)		1(Ref)		1(Ref)	
Q2	1.12 (0.96–1.31)	0.154	1.12 (0.96–1.31)	0.151	1.09 (0.93–1.28)	0.266
Q3	1.16 (0.99–1.36)	0.054	1.16 (0.99–1.36)	0.059	1.07 (0.92–1.26)	0.376
Q4	1.48 (1.27–1.72)	< 0.001	1.48 (1.27–1.73)	< 0.001	1.31 (1.11–1.54)	0.001
P for trend		< 0.001		< 0.001		0.002

Per SD increased of TyG, TyG-BMI, TyG-WC, Ty G-WHtR as continuous variable. SD: standard deviation

Model 1 adjusted for age, sex, marital status, rural

Model 2 adjusted for Model 1 + smoke, drink, WBC, platelet, HGB, cancer, lung disease, stroke, liver disease, renal disease, digestive disease, asthma

Model 3 adjusted for Model2 + LDL, HbA1c, pulse, hypertension, diabetes

In the fully adjusted model (Table 2, model 3), compared with the lowest quartile, those in the highest quartile of TyG-BMI, TyG-WC and TyG-WHtR were significantly associated with a higher risk of new-onset heart disease in all participants, and the adjusted HR (95% CI) were 1.73 (1.47–2.03), 1.46 (1.24–1.71) and 1.31 (1.11–1.54), respectively. In contrast, TyG was not significantly associated with the incidence of heart disease, and the adjusted HR (95% CI) was 1.08 (0.92–1.27).

Further, the mutual adjustment models (Supplementary Material 2: Table S1) of TyG and TyG related indices were performed. The collinearity test showed that TyG-WC and TyG-WHtR were collinearity, so there was no adjustment between TyG-WC and TyG-WHtR. Compared with the lowest quartile, those in the highest quartile of TyG-BMI were significantly associated with the risk of heart disease after adjusted TyG, TyG-WC and TyG-WHtR and the HR (95% CI) is 1.93 (1.55–2.40), which is higher than TyG-WC and TyG-WHtR (Model 4 in Supplementary Material 2: Table S1).

**Fig. 3:**
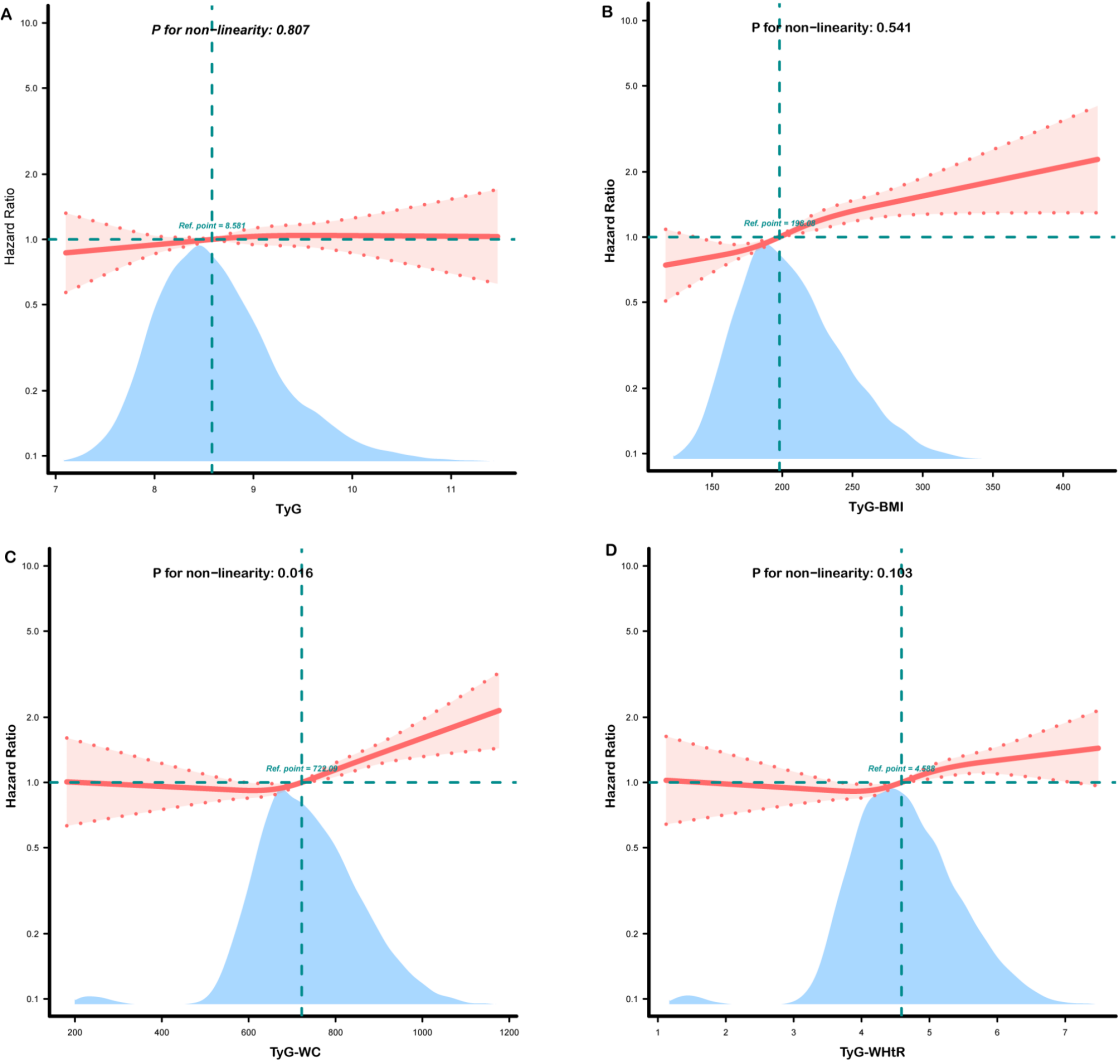
Restricted cubic spline curves for heart disease by TyG (**A**), TyG-BMI (**B**), TyG-WC (**C**), TyG-WHtR (**D**) in all participants after covariate adjustment. Heavy central line represents the estimated adjusted hazard ratio, with shaded ribbons denoting 95% confidence interval. The model is adjusted for age, sex, marital status, rural, smoke, drink, WBC, platelet, HGB, cancer, lung disease, stroke, liver disease, renal disease, digestive disease, asthma, LDL, HbA1c, pulse, hypertension, diabetes

In Fig. 3, we used restricted cubic splines to visualize the associations between TyG, TyG-BMI, TyG-WC, and TyG-WHtR with heart disease events in all participants. After adjusting for all covariates in model 3 of Table 2, a linear correlation was found for TyG, TyG-BMI, and TyG-WHtR (P-nonlinear > 0.05). In contrast, TyG-WC showed nonlinear associations with heart disease (P-nonlinear < 0.05). We further used a two-piecewise model to fit the link between TyG-wc and heart disease risk. As shown in Table 5, when TyG-WC was ≥ 650, the risk of heart disease increased with higher TyG-WC (HR: 1.0015, 95% CI: 1.0008–1.0021). Conversely, when TyG-WC was < 650, increases in TyG-WC did not correlate with increased heart disease risk (HR: 0.9993, 95% CI: 0.9981–1.0004).

**Table 3:** The association of TyG, TyG-BMI, TyG-WC and TyG-WHtR and heart diseas in non-T2D patients

Variable	*n*. %	Without T2D					
		Model 1		Model 2		Model 3	
		HR (95%CI)	*P*	HR (95%CI)	*P*	HR (95%CI)	*P*
TyG	1245 (18.2)	1.08 (1.01–1.15)	0.029	1.05 (0.98–1.12)	0.146	1.04 (0.97–1.12)	0.239
Quartile							
Q1	312 (16.1)	1(Ref)		1(Ref)		1(Ref)	
Q2	333 (18.1)	1.10 (0.94–1.28)	0.237	1.07 (0.91–1.25)	0.404	1.05 (0.9–1.23)	0.532
Q3	344 (19.9)	1.18 (1.01–1.37)	0.04	1.15 (0.98–1.34)	0.088	1.12 (0.96–1.31)	0.163
Q4	256 (19.2)	1.18 (1.01–1.4)	0.048	1.12 (0.94–1.32)	0.205	1.09 (0.92–1.30)	0.304
P for trend			0.026		0.122		0.204
TyG-BMI	1245 (18.2)	1.22 (1.15–1.29)	< 0.001	1.19 (1.13–1.26)	< 0.001	1.18 (1.12–1.26)	< 0.001
Quartile							
Q1	273 (14.5)	1(Ref)		1(Ref)		1(Ref)	
Q2	312 (17.3)	1.26 (1.07–1.48)	0.006	1.27 (1.07–1.49)	0.005	1.26 (1.06–1.48)	0.007
Q3	318 (18.5)	1.35 (1.14–1.59)	< 0.001	1.35 (1.14–1.59)	< 0.001	1.33 (1.12–1.57)	0.001
Q4	342 (23.8)	1.83 (1.55–2.16)	< 0.001	1.75 (1.47–2.08)	< 0.001	1.72 (1.44–2.05)	< 0.001
P for trend			< 0.001		< 0.001		< 0.001
TyG-WC	1245 (18.2)	1.19 (1.11–1.26)	< 0.001	1.16 (1.08–1.23)	< 0.001	1.15 (1.07–1.22)	< 0.001
Quartile							
Q1	281 (14.9)	1(Ref)		1(Ref)		1(Ref)	
Q2	309 (16.9)	1.15 (0.98–1.35)	0.093	1.15 (0.98–1.35)	0.094	1.14 (0.97–1.34)	0.121
Q3	337 (19.5)	1.33 (1.13–1.56)	< 0.001	1.31 (1.12–1.54)	0.001	1.29 (1.1–1.52)	0.002
Q4	318 (22.7)	1.58 (1.35–1.86)	< 0.001	1.47 (1.24–1.75)	< 0.001	1.44 (1.22–1.71)	< 0.001
P for trend			< 0.001		< 0.001		< 0.001
TyG-WHtR	1245 (18.2)	1.15 (1.08–1.23)	< 0.001	1.11 (1.04–1.19)	0.001	1.10 (1.03–1.18)	0.004
Quartile							
Q1	280 (14.9)	1(Ref)		1(Ref)		1(Ref)	
Q2	315 (17.3)	1.14 (0.97–1.34)	0.109	1.13 (0.96–1.32)	0.156	1.12 (0.95–1.32)	0.186
Q3	316 (18.4)	1.17 (1–1.38)	0.056	1.12 (0.95–1.33)	0.166	1.10 (0.93–1.31)	0.246
Q4	334 (23.5)	1.45 (1.23–1.72)	< 0.001	1.34 (1.13–1.60)	0.001	1.31 (1.10–1.57)	0.002
P for trend			< 0.001		0.002		0.005

Per SD increased of TyG, TyG-BMI, TyG-WC, Ty G-WHtR as continuous variable. SD: standard deviation

Model 1 adjusted for age, sex, marital status, rural

Model 2 adjusted for Model 1 + smoke, drink, WBC, platelet, HGB, cancer, lung disease, stroke, liver disease, renal disease, digestive disease, asthma, hypertension

Model 3 adjusted for Model3 + LDL, HbA1c, pulse

**Table 4:** The association of TyG, TyG-BMI, TyG-WC and TyG-WHtR and heart diseas in T2D patients

Variable	*n*. %	With T2D					
		Model 1		Model 2		Model 3	
		HR (95%CI)	*P*	HR (95%CI)	*P*	HR (95%CI)	*P*
TyG	232 (21)	1.01 (0.91–1.12)	0.901	0.97 (0.87–1.08)	0.602	0.98 (0.86–1.12)	0.8
Quartile							
Q1	14 (20.9)	1(Ref)		1(Ref)		1(Ref)	
Q2	31 (23.5)	1.12 (0.6–2.12)	0.717	1.20 (0.63–2.28)	0.571	1.20 (0.63–2.28)	0.586
Q3	42 (16.9)	0.76 (0.41–1.38)	0.364	0.78 (0.42–1.44)	0.423	0.78 (0.42–1.44)	0.43
Q4	145 (22.1)	0.97 (0.56–1.68)	0.908	0.95 (0.54–1.67)	0.857	0.96 (0.54–1.69)	0.883
P for trend			0.852		0.62		0.652
TyG-BMI	232 (21)	1.23 (1.1–1.38)	< 0.001	1.16 (1.02–1.31)	0.019	1.20 (1.06–1.36)	0.005
Quartile							
Q1	13 (12.5)	1(Ref)		1(Ref)		1(Ref)	
Q2	37 (20.1)	1.73 (0.91–3.25)	0.092	1.63 (0.86–3.10)	0.135	1.62 (0.85–3.08)	0.145
Q3	41 (15.2)	1.23 (0.66–2.32)	0.514	1.03 (0.54–1.96)	0.925	1.02 (0.54–1.93)	0.96
Q4	141 (25.7)	2.17 (1.21–3.88)	0.009	1.83 (1.01–3.33)	0.047	1.86 (1.02–3.40)	0.042
P for trend			0.003		0.037		0.027
TyG-WC	232 (21)	1.17 (1.03–1.31)	0.012	1.09 (0.96–1.24)	0.176	1.13 (0.99–1.30)	0.075
Quartile							
Q1	16 (15.4)	1(Ref)		1(Ref)		1(Ref)	
Q2	27 (16.4)	1.14 (0.61–2.12)	0.677	1.19 (0.64–2.23)	0.581	1.16 (0.62–2.19)	0.642
Q3	46 (17.8)	1.19 (0.68–2.11)	0.54	1.06 (0.60–1.89)	0.833	1.04 (0.58–1.84)	0.906
Q4	143 (24.7)	1.69 (1.01–2.83)	0.048	1.49 (0.88–2.52)	0.137	1.51 (0.89–2.57)	0.128
P for trend			0.007		0.059		0.047
TyG-WHtR	232 (21)	1.12 (0.99–1.26)	0.073	1.04 (0.91–1.18)	0.583	1.06 (0.93–1.22)	0.37
Quartile							
Q1	18 (17.1)	1(Ref)		1(Ref)		1(Ref)	
Q2	25 (15.3)	0.90 (0.49–1.65)	0.727	0.81 (0.44–1.49)	0.493	0.83 (0.45–1.53)	0.551
Q3	50 (18.3)	1.02 (0.60–1.76)	0.93	0.84 (0.48–1.45)	0.522	0.82 (0.47–1.42)	0.473
Q4	139 (24.6)	1.35 (0.82–2.23)	0.234	1.08 (0.65–1.80)	0.772	1.11 (0.66–1.86)	0.695
P for trend			0.041		0.281		0.256

Per SD increased of TyG, TyG-BMI, TyG-WC, Ty G-WHtR as continuous variable. SD: standard deviation

Model 1 adjusted for age, sex, marital status, rural

Model 2 adjusted for Model 1 + smoke, drink, WBC, platelet, HGB, cancer, lung disease, stroke, liver disease, renal disease, digestive disease, asthma, hypertension

Model 3 adjusted for Model3 + LDL, HbA1c, pulse

**Table 5:** The non-linearity relationship between TyG-WC and the risk of heart disease

Threshold of waist circumference	HR	95% CI	*P*-value
< 650	0.9993	0.9981,1.0004	0.2089
≥ 650	1.0015	1.0008,1.0021	< 0.001
Non-linear test			0.002

Adjusted for all covariates in Table 2

### The association of TyG and TyG related indices with heart disease in T2D or non-T2D patients

As shown in Table 3, in non-T2D patients, per SD increased of TyG-BMI, TyG-WC and TyG-WHtR were significantly associated with an increased risk of heart disease (TyG-BMI, HR: 1.18, 95% CI: 1.12–1.26, *P* < 0.001; TyG-WC, HR: 1.15, 95% CI: 1.07–1.22, *P* < 0.001; TyG-WHtR, HR: 1.10, 95% CI: 1.03–1.18), whereas TyG was not significantly associated with the incidence of heart disease (HR: 1.04, 95% CI: 0.97–1.12) (Table 3, model 3). However, no significant associations were found between TyG, TyG-WC, TyG-WHtR and heart disease risk in diabetes patients, except for TyG-BMI (HR: 1.20, 95% CI: 1.06–1.36) (Table 4, model 3).

The same association was observed in the categorical variables (Table 3, model 3). When compared with the lowest quartile, the highest quartile of TyG-BMI, TyG-WC and TyG-WHtR were significantly associated with a higher risk of heart disease in patients without diabetes (TyG-BMI, HR: 1.72, 95% CI: 1.44–2.05, *P* < 0.001; TyG-WC, HR: 1.44, 95% CI: 1.22–1.71, *P* < 0.001; TyG-WHtR, HR: 1.31, 95% CI: 1.10–1.57), but TyG was also not significantly associated with the incidence of heart disease (HR: 1.09, 95% CI: 0.92–1.30) in non-T2D patients (Table 3, model 3). However, no significant associations were found between TyG, TyG-WC, TyG-WHtR and heart disease risk in diabetes patients, except for the highest quartile of TyG-BMI (HR: 1.86, 95% CI: 1.02–3.40) (Table 4, model 3).

### Subgroup analysis and sensitivity analysis

**Fig. 4:**
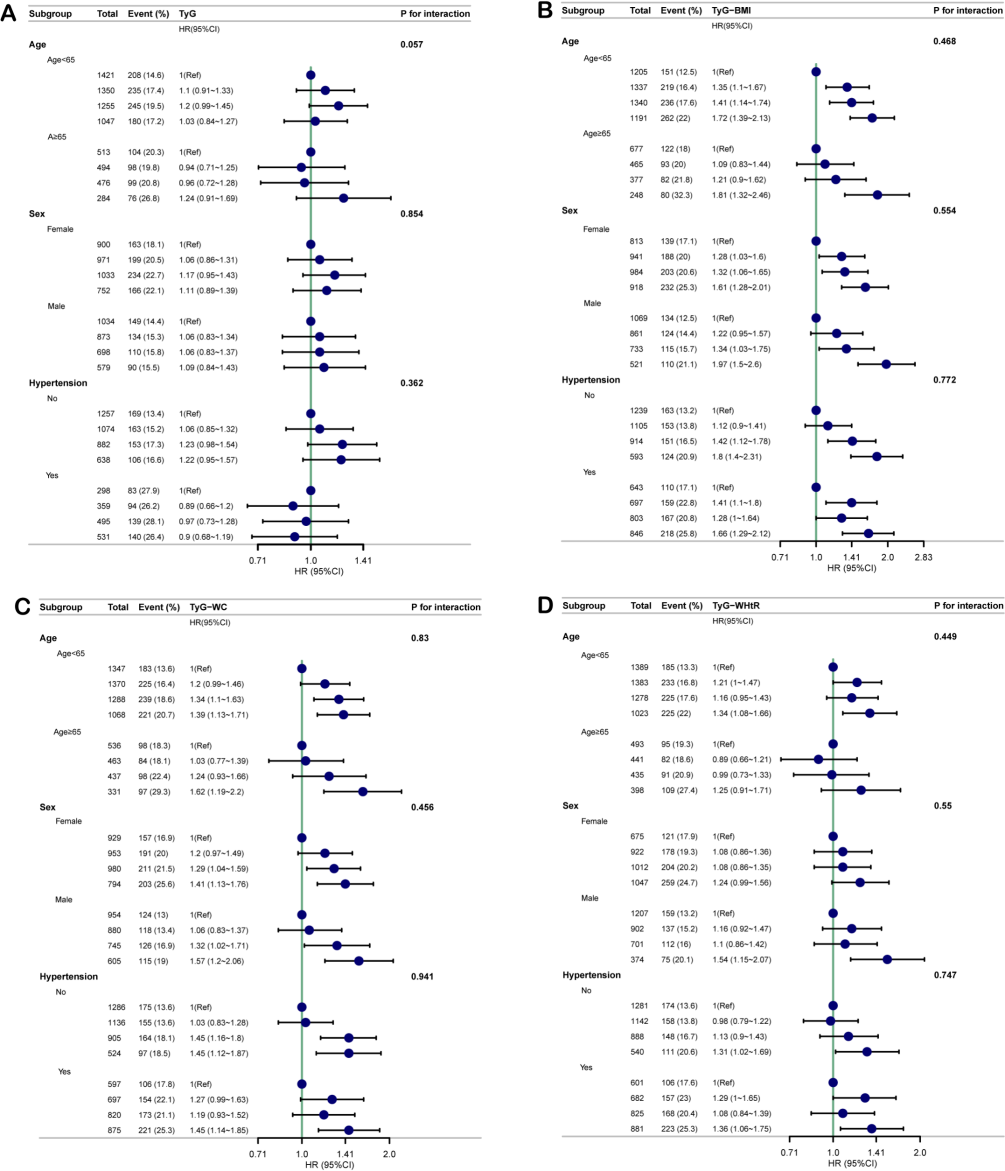
Subgroup analyses in non-diabetic population for the association of TyG (**A**), TyG-BMI (**B**), TyG-WC (**C**) and TyG-WHtR (**D**) with risk of heart disease in age, sex, hypertension subgroup. The HR was calculated using Cox proportional hazards model with the adjustments including age, sex, marital status, rural, smoke, drink, WBC, platelet, HGB, cancer, lung disease, stroke, liver disease, renal disease, digestive disease, asthma, LDL, HbA1c, pulse, hypertension. The P values for the interaction effect that using the likelihood ratio test are annotated to the right of **A**, **B**, **C**, and **D**. HR hazard ratio, CI confidence interval

As shown in Fig. 4, we further analyzed the HR effect sizes of TyG (Fig. 4A), TyG-BMI (Fig. 4B), TyG-WC (Fig. 4C), and TyG-WHtR (Fig. 4D) on heart disease in non-T2D patients across subgroups of age (< 65 years and ≥ 65 years), sex, and hypertension status. The P values for interaction effects, derived from the likelihood ratio test, are indicated to the right of each figure. No statistically significant interaction effects were observed in the age, sex, and hypertension subgroups (all P for interaction > 0.05). Furthermore, E-value were tested for the influence of unmeasured confounders as a sensitivity analysis. With E-value much larger than HR value, we found that unmeasured confounding is unlikely to influence our results (Supplementary Material 3: Table S2).

## Discussion

In this nationwide cohort study, our results demonstrated that TyG-BMI, TyG-WC and TyG-WHtR were associated with an increased risk of heart disease. Furthermore, we explored the relationship between TyG, TyG related indices and heart disease in people with and without diabetes. Indeed, we found that the risk of heart disease increased with higher levels of TyG-BMI, TyG-WC, and TyG-WHtR in individuals without diabetes. However, in the diabetic population, the association was not significant, with the exception of TyG-BMI, which needs further investigation [19–21]. The results of subgroup analysis showed that TyG-BMI and TyG-WC were relatively stable in association to heart disease among all subgroups. In addition, higher hazard ratios were shown in the male group of TyG-BMI, TyG-WC, and TyG_WHtR, which may be associated with higher abdominal fat accumulation and cardiovascular risk in male. These findings suggest that TyG combined with obesity indicators may be more effective in the national male population. These findings suggest that TyG combined with obesity indicators may be more effective in the Chinese population (Table 5).

The accuracy of the TyG and TyG related indices in assessing insulin resistance varies depending on the ethnic and cultural context. Our study observed that TyG-BMI may be the strongest association with cardiovascular risk in national population, while TyG alone does not appear to be applicable in this study population. We also found a nonlinear relationship between TyG-WC and heart disease. A study based on a large population in the United States from NHANSE database found that TyG-WC had the highest correlation with total cardiovascular disease and TyG-BMI was more effective than BMI in diagnosing CVD and CVD mortality [22]. Results from a study involving 1,145 Korean subjects also showed that TyG-WC was more effective than TyG and TyG-BMI in diagnosing the progression of coronary artery calcification [23]. Another study based on a UK population found an independent association between the TyG index and the risk of aortic dissection and aneurys [24]. TyG, TyG related indices show differences in cardiovascular risk associations among populations of different races and regions, and this study provides some evidence in this field to some extent.

Our study found that TyG-BMI, TyG-WC, and TyG-WHtR are associated with cardiovascular risk in the national population, and this association was observed in non-T2D participant but not found in T2D patients, except for TyG-BMI. Because of the samples of T2D patients, we can’t conclude any statistical results. But reviewing previous studies, we found that Laura Sánchez-Íñigo et al. [25] and Liu Li et al. [4] were consistent with our findings. This may be related to glucose-lowering medication in diabetic patients, affecting the blood glucose level and directly influencing the TyG index. In addition, in the diabetic population, traditional cardiovascular risk factors have a more significant impact on cardiovascular events than IR [22]. Another studies also point out that insulin resistance itself is not a major risk factor for coronary heart disease in people with diabetes. According to one study, Men with Type 2 diabetes were more likely to have multiple risk factors and higher levels of haemostatic and inflammatory markers than men without, irrespective of CHD status. Compared with men with CHD only, men with diabetes and CHD showed increased levels of tissue plasminogen activator antigen, increased plasma and blood viscosity, and increased levels of coagulation factors VII, VIII and IX [26].

Despite the lack of consensus on the mechanisms underlying the relationship between TyG, TyG related indices and the risk of heart disease, several hypotheses can be proposed. The TyG index (triglyceride-glucose index) and its derivatives TyG-BMI, TyG-WC and TyG-WHtR are all indicators of IR assessment [27–29]. IR plays a role in the onset and progression of cardiovascular disease through a number of mechanisms [30]. For example, IR results in disorders of lipid metabolism, affects high-density lipoprotein cholesterol levels, stimulates the secretion of inflammatory factors by endothelial cells, and accelerates the progression of atherosclerosis [31]. Furthermore, IR results in cardiomyocyte damage, myocardial fibrosis and myocardial hypertrophy, thereby increasing the risk of heart failure [32]. IR is also closely associated with metabolic syndrome (MetS), which is an important risk factor for cardiovascular diseas [33]. In China, the incidence of metabolic syndrome and its associated cardiovascular diseases is rapidly increasing in conjunction with rapid economic development and lifestyle changes [34].

The present study has several key strengths that contribute to its significance in the field. Firstly, the study benefits from a large sample size, extensive coverage, and the representativeness of the national population [35]. But it is important to recognize the limitations of the present study. Firstly, In the CHARLS database, several important covariates were missing: retinopathy, neuropathy, family history of CVD, pulse, medication. Secondly, despite efforts to adjust for relevant confounders in the multivariate model, the possibility of unmeasured or unknown residual confounders cannot be entirely discounted. These factors may have potentially led to an overestimation or underestimation of the observed associations. However, given the reasonably adjustment for several significant confounding factors and assessment of E-values for unmeasured confounders, any impact on the overall results due to the absence of certain variables is likely to be minimal. Thirdly, this study did not adjust for time-dependent variables, The relationship between time-dependent indicators and heart disease needs to be further explored in the future.

## Conclusions

In the national population study, higher TyG-BMI, TyG-WC, and TyG-WHtR indices were significantly associated with an increased risk of heart disease, with TyG-BMI and TyG-WC showing stronger correlations. While this association was evident in non-T2D patients, only TyG-BMI was associated with heart disease incidence in T2D patients, underscoring the necessity for further investigation.

## Electronic supplementary material

Below is the link to the electronic supplementary material.

Supplementary Material 1: Figure S1 Kaplan-Meier survival analysis curves of heart disease risk by TyG (A), TyG-BMI (B), TyG-WC (C), TyG-WHtR (D) in all participants. TyG: Triglyceride and glucose, TyG-BMI: Triglyceride and glucose with body mass index, TyG-WC: Triglyceride and glucose with waist circumference, TyG-WHtR: Triglyceride and glucose with waist-to‐height ratio.

Supplementary Material 2

Supplementary Material 3

## Data Availability

All data in the article were obtained from the CHARLS database (https://charls.pku.edu.cn/).
