# Correction: The association of triglyceride-glucose and triglyceride-glucose related indices with the risk of heart disease in a national cohort study

**DOI:** 10.1186/s12933-025-02726-4

**Published:** 2025-04-19

**Authors:** Xiaodi Tang, Kexin Zhang, Rong He

**Affiliations:** Department of Cardiovascular Medicine, Beijing Tsinghua Changgung Hospital, School of Clinical Medicine, Tsinghua University, No. 168 Litang Road, Changping District, Beijing, China

**Correction to: Cardiovascular Diabetology (2025) 24:54** 10.1186/s12933-025-02621-y

In the original publication of this article [1], the title was incorrectly given as ‘The association of triglyceride-glucose and triglyceride-glucose related indices with the risk of heart disease in a national’ but should have been ‘The association of triglyceride-glucose and triglyceride-glucose related indices with the risk of heart disease in a national cohort study’.

The original article has been corrected.
