# Supplementary figures and images for: The association of triglyceride-glucose and triglyceride-glucose related indices with the risk of heart disease in a national cohort study
Source: Cardiovasc Diabetol. 2025 Feb 6;24:54. doi: 10.1186/s12933-025-02621-y (PMC11803996; doi:10.1186/s12933-025-02621-y)

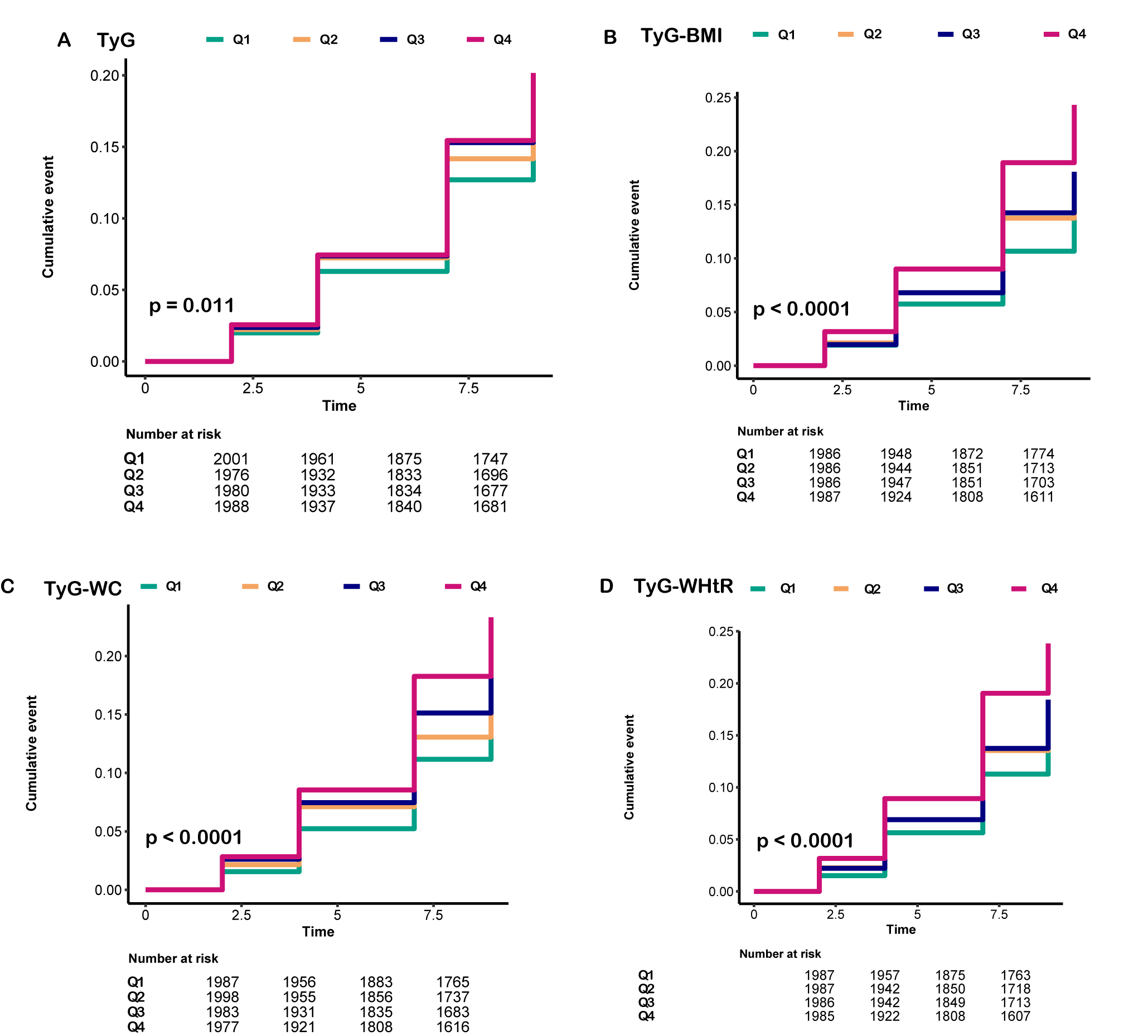

Supplement: Supplementary file 1 — Supplementary Material 1: Figure S1 Kaplan-Meier survival analysis curves of heart disease risk by TyG (A), TyG-BMI (B), TyG-WC (C), TyG-WHtR (D) in all participants. TyG: Triglyceride and glucose, TyG-BMI: Triglyceride and glucose with body mass index, TyG-WC: Triglyceride and glucose with waist circumference, TyG-WHtR: Triglyceride and glucose with waist-to‐height ratio. [file 12933_2025_2621_MOESM1_ESM.tif]
