# Supplementary material for: The association of triglyceride-glucose and triglyceride-glucose related indices with the risk of heart disease in a national cohort study
Source: Cardiovasc Diabetol. 2025 Feb 6;24:54. doi: 10.1186/s12933-025-02621-y (PMC11803996; doi:10.1186/s12933-025-02621-y)
Supplement: Supplementary file 2 — Supplementary Material 2 [file 12933_2025_2621_MOESM2_ESM.docx]

**Table S1.1** The association between TyG-BMI and heart diseas in all participants.

| Variable | **Model 1** | |  | **Model 2** | |  | **Model 3** | |  | **Model 4** | |
| --- | --- | --- | --- | --- | --- | --- | --- | --- | --- | --- | --- |
|  | **HR (95%CI)** | **P value** |  | **HR (95%CI)** | **P value** |  | **HR (95%CI)** | **P value** |  | **HR (95%CI)** | **P value** |
| TyG-BMI | 1.04 (1.02 - 1.06) | <0.001 |  | 1.03 (1.01 - 1.05) | 0.001 |  | 1.04 (1.01 - 1.06) | <0.001 |  | 1.24 (1.16 - 1.33) | <0.001 |
| Quartile |  |  |  |  |  |  |  |  |  |  |  |
| Q1 | 1(Ref) |  |  | 1(Ref) |  |  | 1(Ref) |  |  | 1(Ref) |  |
| Q2 | 1.28 (1.09 - 1.49) | 0.002 |  | 1.30 (1.11 - 1.52) | <0.001 |  | 1.26 (1.08 - 1.48) | 0.004 |  | 1.32 (1.12 - 1.55) | 0.001 |
| Q3 | 1.32 (1.13 - 1.54) | 0.001 |  | 1.36(1.16 - 1.59) | <0.001 |  | 1.27 (1.08 - 1.5) | 0.003 |  | 1.36 (1.13 - 1.64) | 0.001 |
| Q4 | 1.88 (1.61 - 2.18) | <0.001 |  | 1.92 (1.64 - 2.24) | <0.001 |  | 1.75 (1.49 - 2.06) | <0.001 |  | 1.93 (1.55 - 2.40) | <0.001 |
| P for trend |  | <0.001 |  |  | <0.001 |  |  | <0.001 |  |  | <0.001 |

Per SD increased of TyG-BMI as continuous variable. SD: standard deviation.

Model 1 adjusted for age, sex, marital status, rural.

Model 2 adjusted for Model 1 + smoke, drink, WBC, platelet, HGB, cancer, lung disease, stroke, liver disease, renal disease, digestive disease, asthma.

Model 3 adjusted for Model2 + LDL, HbA1c, pulse, hypertension, diabetes.

Model 4 adjusted for Model3 + TyG, TyG-WC and TyG-WHtR.

**Table S1.2** The association between TyG-WC and heart diseas in all participants.

| Variable | **Model 1** | |  | **Model 2** | |  | **Model 3** | |  | **Model 4** | |
| --- | --- | --- | --- | --- | --- | --- | --- | --- | --- | --- | --- |
|  | **HR (95%CI)** | **P value** |  | **HR (95%CI)** | **P value** |  | **HR (95%CI)** | **P value** |  | **HR (95%CI)** | **P value** |
| TyG-WC | 1.04 (1.02 - 1.06) | <0.001 |  | 1.03 (1.01 - 1.05) | 0.001 |  | 1.14 (1.08 - 1.21) | <0.001 |  | 1.06 (0.98 - 1.15) | 0.157 |
| Quartile |  |  |  |  |  |  |  |  |  |  |  |
| Q1 | 1(Ref) |  |  | 1(Ref) |  |  | 1(Ref) |  |  | 1(Ref) |  |
| Q2 | 1.28 (1.09 - 1.49) | 0.002 |  | 1.30 (1.11 - 1.52) | <0.001 |  | 1.12 (0.96 - 1.31) | 0.156 |  | 1.08 (0.92 - 1.26) | 0.375 |
| Q3 | 1.32 (1.13 - 1.54) | 0.001 |  | 1.36(1.16 - 1.59) | <0.001 |  | 1.24 (1.06 - 1.45) | 0.006 |  | 1.12 (0.94 - 1.34) | 0.20 |
| Q4 | 1.88 (1.61 - 2.18) | <0.001 |  | 1.92 (1.64 - 2.24) | <0.001 |  | 1.46 (1.24 - 1.71) | <0.001 |  | 1.20 (0.96 - 1.49) | 0.117 |
| P for trend |  | <0.001 |  |  | <0.001 |  |  | <0.001 |  |  | 0.116 |

Per SD increased of TyG-WC as continuous variable. SD: standard deviation.

Model 1 adjusted for age, sex, marital status, rural.

Model 2 adjusted for Model 1 + smoke, drink, WBC, platelet, HGB, cancer, lung disease, stroke, liver disease, renal disease, digestive disease, asthma.

Model 3 adjusted for Model2 + LDL, HbA1c, pulse, hypertension, diabetes.

Model 4 adjusted for Model3 + TyG, TyG-BMI.

**Table S1.3** The association between TyG-WHtR and heart diseas in all participants.

| Variable | **Model 1** | |  | **Model 2** | |  | **Model 3** | |  | **Model 4** | |
| --- | --- | --- | --- | --- | --- | --- | --- | --- | --- | --- | --- |
|  | **HR (95%CI)** | **P value** |  | **HR (95%CI)** | **P value** |  | **HR (95%CI)** | **P value** |  | **HR (95%CI)** | **P value** |
| TyG-WHtR | 1.04 (1.02 - 1.06) | <0.001 |  | 1.03 (1.01 - 1.05) | 0.001 |  | 1.07 (1.03 - 1.10) | <0.001 |  | 0.98 (0.91 - 1.05) | 0.556 |
| Quartile |  |  |  |  |  |  |  |  |  |  |  |
| Q1 | 1(Ref) |  |  | 1(Ref) |  |  | 1(Ref) |  |  | 1(Ref) |  |
| Q2 | 1.28 (1.09 - 1.49) | 0.002 |  | 1.30 (1.11 - 1.52) | <0.001 |  | 1.10 (0.94 - 1.29) | 0.238 |  | 1.02 (0.87 - 1.2) | 0.834 |
| Q3 | 1.32 (1.13 - 1.54) | 0.001 |  | 1.36(1.16 - 1.59) | <0.001 |  | 1.08 (0.92 - 1.26) | 0.355 |  | 0.92 (0.77 - 1.09) | 0.324 |
| Q4 | 1.88 (1.61 - 2.18) | <0.001 |  | 1.92 (1.64 - 2.24) | <0.001 |  | 1.33 (1.13 - 1.57) | 0.001 |  | 0.95 (0.76 - 1.18) | 0.637 |
| P for trend |  | <0.001 |  |  | <0.001 |  |  | 0.001 |  |  | 0.423 |

Per SD increased of TyG-WHtR as continuous variable. SD: standard deviation.

Model 1 adjusted for age, sex, marital status, rural.

Model 2 adjusted for Model 1 + smoke, drink, WBC, platelet, HGB, cancer, lung disease, stroke, liver disease, renal disease, digestive disease, asthma.

Model 3 adjusted for Model2 + LDL, HbA1c, pulse, hypertension, diabetes.

Model 4 adjusted for Model3 + TyG, TyG-BMI.

Table S4 Test of collinearity with TyG, TyG-BMI, TyG-WC and TyG-WHtR.

| **Term** | **VIF** | **colinearity** |
| --- | --- | --- |
| TyG | 1.852 | 0 |
| TyG-BMI | 2.423 | 0 |
| TyG-WC | 11.596 | 1 |
| TyG-WHtR | 10.932 | 1 |
