# Supplementary material for: The association of triglyceride-glucose and triglyceride-glucose related indices with the risk of heart disease in a national cohort study
Source: Cardiovasc Diabetol. 2025 Feb 6;24:54. doi: 10.1186/s12933-025-02621-y (PMC11803996; doi:10.1186/s12933-025-02621-y)
Supplement: Supplementary file 3 — Supplementary Material 3 [file 12933_2025_2621_MOESM3_ESM.docx]

**Table S2.1** E-value between TyG index, TyG related indices and heart disease in all patients.

| **Variable** | Item | point | lower | upper |
| --- | --- | --- | --- | --- |
| TyG-BMI | RR | 1.73 | 1.47 | 2.03 |
|  | E-values | 2.85 | 2.30 | nan |
| TyG-WC | RR | 1.46 | 1.24 | 1.71 |
|  | E-values | 2.28 | 1.79 | nan |
| TyG-WHtR | RR | 1.31 | 1.11 | 1.54 |
|  | E-values | 1.95 | 1.46 | nan |

RR: relative Risk

**Table S2.2** E-value between TyG index, TyG related indices and heart disease in non-diabetes patients.

| **Variable** | Item | point | lower | upper |
| --- | --- | --- | --- | --- |
| TyG-BMI | RR | 1.72 | 1.44 | 2.05 |
|  | E-values | 2.83 | 2.24 | nan |
| TyG-WC | RR | 1.44 | 1.22 | 1.71 |
|  | E-values | 2.24 | 1.74 | nan |
| TyG-WHtR | RR | 1.31 | 1.10 | 1.57 |
|  | E-values | 1.95 | 1.43 | nan |

RR: relative Risk
